# Organic form and evolution: the morphological problem in twentieth-century italian biology

**DOI:** 10.1007/s40656-022-00534-7

**Published:** 2022-11-03

**Authors:** Marco Tamborini

**Affiliations:** Institut für Philosophie, Technische Universität Darmstadt, Residenzschloss 1, 64283 Darmstadt, Germany

## Abstract

This paper examines the efforts in evolution research to understand form’s structure that developed in Italy during the first half of the twentieth century. In particular, it analyzes how the organic approach in biology and the study of organic form merged in the morphological research agendas of Giuseppe Colosi (1892–1975) and Giuseppe Levi (1872–1965). These biologists sought to understand form’s inner composition and structure. First, I will briefly outline the morphological practices and frameworks used to study form changes and structures in the early twentieth century. Second, I will discuss what the Italian biologist Antonio Pensa (1874–1970) called the morphological problem. Third, I will examine Colosi’s response to the morphological problem. Fourth, I will analyze Levi’s morphological research program. As a result, this paper paves the way for a more nuanced and varied picture of the so-called “organicism movement” in the first half of the twentieth century by calling attention to morphology as practiced in Italian-speaking biology. In fact, alongside dialectical materialism and holistic biology, two of the main strands within organicism, the architectural approach to evolution as practiced in Italy and elsewhere had a profound impact on twentieth- and twenty-first-century organicism specifically and on evolutionary biology generally.

In 1910 at the Eighth International Physiological Congress in Vienna, the French physiologist and Nobel laureate Charles Richet (1850–1935) lamented the uselessness of morphology for genuine biological investigations. The French physiologist denounced the descriptive study of organic forms, asserting that they did not broaden understandings of how particular organs work or the chemical and physiological processes therein (Circular, 1910). At the beginning of the twentieth century, Richet’s ideas were quite widespread. The place of morphology within the life-sciences-system of knowledge was carefully debated and scrutinized. For instance, as the British biologist Edward Stuart Russell (1887–1954), in his seminal book *Form and Function* (1916), asserted that the entire history of morphology could be narrated by drawing on the two quite opposing categories of form and function. In the concluding remarks, Russell professed that the coming decades “will see a return to a simpler and more humble attitude towards the great and unsolved problems of animal form” (Russell, 1916, p. 364). However, the majority of scientists shared Richet’s view. They understood morphology as a descriptive discipline, distinct from the growing bulk of knowledge produced by the experimental sciences. Within this panorama, Italian biologist Giuseppe Levi (1872–1965) reacted quite sharply to Richet and colleagues’ insinuations on the usefulness of morphology for biological investigations. He deemed Richet’s judgment “crude” and even dangerous, given Richet’s position of influence (Levi, 1920, p. 38). “Even in the past”, commented Levi, “we have witnessed an alternating affair of enthusiasm and discouragement on the importance of the morphological address in Biology, and the science of forms, the oldest of all, renewing itself in opportune times, was never submerged and will not be, because it cannot be separated from [the science] of life” (ibid.). In response, Levi published several works with the intention of “rebut[ting] such claims of failure of the morphological method” (ibid., p. 40). In particular, he sought to use morphology to uncover broader biological mechanisms and evolutionary phenomena.

By investigating the practices used by Italian biologists^1^ to analyze morphological changes, this paper examines the evolutionary research efforts to understand form’s structure developed in Italy during the first half of the twentieth century. Specifically, it analyzes how the organicism approach to biology and the study of organic form fused in the morphological research agendas of Giuseppe Colosi (1892–1975) and Giuseppe Levi. These scientists were influenced by German-speaking and Anglophone scientists, particularly by the advocates of the so-called organicism biology^2^. At the same time, they developed their own research agenda and practices to explore the dynamics of form structures. Specifically, they sought to understand the inner composition and structure of form. They explored the possibility of form change, in which natural selection and chance play a minor role. The morphological investigations of the inner structure of form conducted by Colosi and Levi were shared with several international biologists, including Russian zoologists Michael M. Nowikoff (1876–1965) and Lev Semyonovich Berg (1876–1950). These scientists networked and proposed an approach to morphology and evolution, which I have defined somewhere else as the “the architectural approach to form” (Tamborini 2022b).

This article will provide new material that can be used to reconsider the contributions of Italian biologists to twentieth-century evolutionary theory and reexamine the debate on the role of morphology during that timeframe. This will help expand our understanding of some of the central categories featured in the history of early twentieth-century biology such as the notions of mechanism, organicism, and vitalism^3^.

Evolutionary research has often been considered a quite marginal issue in twentieth-century Italian-speaking biology. This was because Italian biologists accepted Mendel’s laws and Morgan’s chromosome theory of heredity rather late, and they did not contribute at all to the development of the Modern Synthesis of Evolution. Despite this delay, innovative evolutionary investigations were indeed conducted in Italy. Biologists sought to understand the notion of organic form, its development, and its changes through time. By doing so, they wanted to uncover new and non-Darwinian evolutionary mechanisms as Colosi, following his teacher Daniele Rosa, sought to.

This paper will first briefly depict the morphological practices and frameworks used to investigate form changes and structures at the beginning of the twentieth century. Second, I will tackle what has been called by Italian anatomist Antonio Pensa (1874–1970) the morphological problem. In a nutshell, practitioners asked for the grounds behind the appearance of a series of regular body plans that seem to be found in diverse families despite form great variations. Third, I will examine Colosi’s answer to the morphological problem. Building his practices on the theory of *Hologenesis* as developed by his former teacher Daniele Rosa, he focused his analysis on the morphological analysis of parallelism. I will then move to Levi’s morphological research agenda. He expanded on the morphological tradition proper of Scottish polymath D’Arcy Thompson and Austrian biologist Hans Leo Przibram (1874–1944) and called attention to, among other things, the dynamics of growth and aging in organisms. In the conclusion, I will reflect on the broader features of Italian morphology^4^ and connect it to the organicism movement of the early twentieth century.

## Morphology at the beginning of the twentieth century

Paradoxically, at the beginning of the twentieth century, morphological research was both decreasing in its prestige and expanding across different disciplines^5^. The methods and approaches used to grasp the dynamics of form changes grew exponentially during this time. As in all western countries host to this type of scientific research, in Italy, three different approaches were defended and developed.

Vitalism, the strongest approach to the study of form in Italy, was led by Eugenio Rignano (1870–1930). Vitalists sought to understand form changes as an autonomous and independent phenomenon. Life, they claimed, cannot be reduced to chemico-physical forces, nor can it be fully explained by the Darwinian theory of evolution. Natural selection and chance, they argued, do not have the power to produce perfected and well-adapted forms. Rather, they asserted the existence of an intrinsic principle in the organism responsible for its full development. Following Aristoteles, German biologist Hans Driesch (1867–1941) named this principle “entelechy”. He argued that organisms had the inherent vital component, entelechy, that caused them to develop and rebuild themselves^6^.

A further issue addressed by vitalists was that of organisms’ own historicity. In what way, some biologists wondered, does the evolutionary past determine the specificity of organic form? A living being is related to the past by a link comparable to that of psychic memory. In this regard, Rignano spoke, for instance, of a sort of mnemonic property of the living material. He supported the hypothesis that there was a particular form of energy specific to living beings: the nervous mnemonic energy. This type of energy, similar to energy in the inorganic world, has the property of specific accumulation. This means that can accumulate (as electricity does in an accumulator) in the nuclei of vital cells and then discharge the same energy that caused the accumulation (Rignano, 1926). As a result of these positions, Rignano’s theories were debated internationally, finding both supporters and opponents.

The second approach to morphology and broader evolution features a strong approach. This approach to the organism and the development of form was characterized by both a methodological and ontological reductionism between the organism and the characteristics of the machine. Indeed, the organism was compared to and sometimes identified as a machine. The understandings of the physical, thermodynamic, and chemical transformation principles of matter were identified as the fundamental traits to understand the form, structure, and functioning of an organism. Famously, the German-American physiologist Jacques Loeb (1859–1924) argued for a complete ontological identity between machines and organisms^7^. In his 1912 seminal book *The Mechanistic Conception of Life*, he even argued that “not only is the mechanistic conception of life compatible with ethics; it seems the only conception of life which can lead to an understanding of the source of ethics” (Loeb, 1912, p. 51).

The third approach was called organicism. It was identified by a strong denial of some extremist components of both vitalism and mechanism. By doing so, its supports identified themselves as proponents of a “third way” which was neither vitalism nor mechanism. The organism and the organic form have a kind of autonomy with respect to the inorganic world. This, however, does not mean that the maintenance of form was attributable to metaphysical forces. On the contrary, this autonomy was detectable by treating the organisms. The characteristics of the organicist movement have been extensively analyzed. Following Jan Baedke and others (Harrington, 1999; Baedke, 2019; Nicholson & Gawne, 2015), they are reducible to three basic pillars: (i) neither mechanism nor vitalism, (ii) organism as the main theoretical concept for biology, and (iii) the study of an organism as a whole.

Alongside these three strands, a fourth undertaking cut across the interests and methods of the first three. The crux of the matter for these biologists was defined by Italian anatomist Antonio Pensa as “the morphological problem”. With this, he meant that biologists were struggling to understand the inner architectural composition of organic form. This comprehension would enable them to grasp the laws supporting morphogenesis. The rationale was to identify and illustrate the building principles responsible for producing and developing stable organic forms. In Italian-speaking biology, the main figures in this fourth approach to were Italian biologists Giuseppe Colosi and Giuseppe Levi. However, before dealing with Colosi’s and Levi’s practices, we should focus on the morphological problem posed by Pensa.

## The morphological problem

Italian Anatomist Antonio Pensa taught anatomy, embryology, and cytology in Sassari, Padua, and Parma. In one of his lectures to the students of human anatomy in Parma, he dedicated several pages to the main epistemological issue, which was meant to unify the whole study of the structure of organisms and their parts. He named it “the morphological problem”. Pansa presented this problem through a quite simple and plain question: “Why and how does it happen that organized beings present on the one hand a general plan of form and structure and on the other hand those features which are characteristic of each individual species and which are transmitted, with admirable regularity from generation to generation?” (Pensa, 1922, p. 487).

Although the morphological problem has been considered in almost all disciplines concerned with the study of living things, Pensa promptly found that scholars in a range of fields had not addressed the problem sufficiently. Histology seemed to take an appropriate approach, but even the most promising histological analyses turned up nothing. According to Pensa, the same could be said for Wilhelm Roux’s mechanical morphology: “despite the great light [mechanical morphology] brings to the knowledge of the nexus between structure and function, it does not solve the morphological problem” (ibid.). The morphological problem, continued Pensa, could only be addressed if the study of form would move from a merely descriptive classification of shapes to a meticulous search for its “internal causes” of production and variation. At first glance, the reference to the search for internal causes may seem to be an appeal to orthogenetic and vitalist theories. However, Pensa had a quite different methodology in mind to fully address the morphological problem: a structural and architectural approach derived from the use of the microscope. As he put it, “the microscopic examination shows us what aspect the protoplasm has and we call this aspect structure. But if the protoplasm has a structure, it is logical to admit that it represents the architectural foundation of the form of organized beings and that the establishment of what is determined by that structure leads to the solution of the morphological problem” (ibid., p. 495).

Thus, Pensa declined the framing of the morphological problem as an architectural-structural issue. He shifted the phylogenetic study of form from the dialectical opposition between morphological typology and its variation to the level of the constituent elements of form. He first focused on the protoplasm. This shift was quite pragmatic since it allowed him to identify a basic and well-defined unit from which to study the organizational principles responsible for form change and stability. “One of the fundamental characters of the protoplasm that above all characterizes it”, wrote the Italian biologist, “is this: to be always individualized in circumscribed masses and delimited in space, of microscopic dimensions” (ibid., p. 495).

Pensa’s main concern was, therefore, the analysis of the dynamics of form changes. In fact, the morphological study showed that if we observed and took into account comparative data, if the morphologists would analyze the cell in all its vital states and follow them in the various moments of possible functional expressions, “we see that cytoplasm and karyoplast are polymorphic, in the sense that they present various aspects depending on the various types of cells and depending on the various functional moments of them. This property of the protoplasm to change its morphological appearance in relation to function constitutes the morphological metabolism” (ibid., p. 500).

From these architectural-structural analyses, Pensa brought out more general regularities regarding the characterizing elements of form and the difference between crystals and organisms.^8^ Since protoplasm was essentially composed of colloidal substances, the analysis of the conditions by which colloidal systems were composed was indispensable for studying the architecture of forms. Pensa, therefore, noted that “the exact and profound knowledge of what takes place in colloidal substances in general, will contribute much to provide us with the documents for the solution of the morphological problem” (ibid., p. 508).

To reach this goal, it was crucial to establish a deep collaboration between morphologists and chemists.^9^ Pensa added, however, that collaboration among scientists does not imply a principle of subordination between disciplines that could lead to a hierarchical arrangement of sciences under the dominance of chemistry. “Collaboration or, worse, limiting ourselves to borrowing from chemico-physics, [whose data seems] to agree with the morphological facts that we want to explain, are not enough. Chemico-physics must become part of the scientific education program of the morphologist. It is too little to make microscopic preparations and describe their appearances as structural facts. One must interpret these appearances” (ibid.).

In the first half of the twentieth century, the possible architectural composition of form and the morphological problem would become central to Italian biology. One of the biologists who declined it in an evolutionary (and anti-Darwinian) manner was Giuseppe Colosi. With this move, he became part of a network of biologists interested in the evolutionary study of the principles that hold together and regulate the internal organization of forms.

## Form, architecture, and evolution

Although nearly forgotten today, Colosi was one of the main figures of the architectural and structural analysis of organic form during the twentieth century – the so-called analysis of the architecture of organisms. Colosi studied with Italian zoologist Daniele Rosa (1857–1944) in both Florence and Turin. After several lectureships in Turin, Padua, Napoli, Siena, and Pisa, he became the Head of the Zoological Institute at the University of Florence from 1940 to 1962. In his research, the Italian biologist defended a strong aversion to a purely genetic and causal explanation of morphogenesis. Conversely, he studied the anatomy of several forms of *Euphausiacea* crustaceans and took note of important morphological constants. In several subfamilies, several topological relationships were traceable between the legs of the second thoracic pair of *Thysanoessa* and *Tessarabrachion* and those of the third pair of *Stylocheisen*. These parallels, according to Colosi, showed the “relative scarcity of elementary characters compatible with life and their maximum utilization, so that the multiplicity of the organized forms is a consequence of the various grouping of morphological possibilities rather than the appearance each time of new elementary characters” (Colosi, 1921, p. 44). The study of the morphological parallels between different organisms paved the way for Colosi to reflect on the composition and available materials essential for shaping organisms’ form. Therefore, he insisted that:

When studying the complexity of living beings, we are certainly surprised by the diversity of their forms and architectures; however, the analysis of their construction should not be less interesting for the relatively limited number of building materials and the relatively small number of elementary architectural devices. The diversity of life forms therefore seems to depend essentially on the different combinations of building materials and elementary architectural devices in life-compatible total systems.^10^ (Colosi, 1925, p. 313)

Thus, the study of morphological parallelism and, more broadly, the investigation of the phenomena of morphological analogy could help comparative morphology “take a big step forward avoiding falling into the easy mistake of assuming one or the other organic resemblance as proof of this or that phylogenetic derivation” (Colosi 1930, p.265). Only through a serious study of all of the classifiable analogies could biologists discover “the laws that regulate the occurrence of similar structures in the various Phyla of the organized world” (ibid.).

Starting from these empirical results, Colosi proclaimed a much more general conclusion:

no morphological character presented by any species and considered individually can be considered exclusive to that species […] the multiplicity of living forms depends essentially on the different combination of building materials and morphological devices compatible with life instead of the independent appearance of new characters case by case. [We can add] that in the realization of the forms of a group, conformations and characters already found in the other systematic groups are repeated all the more easily and more easily as their kinship is closer, and that the repetitions of the same character in the different species take place according to a rule. (Colosi, 1940, p. 103)

Second, the structural development identified was only possible “through a process due to internal causes” (Colosi, 1928, p. 268). From this assertion, Colosi revealed his intellectual debt to his former teacher, Italian biologist Daniele de Rosa. Rosa deeply admired Colosi in return. In both public and private correspondence, he identified Colosi’s investigations as a solid extension of his own research agenda.

Rosa was a controversial figure in early twentieth-century biology. He based his theoretical reflection upon a strong parallel between the life of species and the life of individuals. Given this parallelism, Rosa reconstructed possible phylogenetic trees:

from each mother-species, two same species of daughters are formed (pre-determined by the structure of the mother species) and the phenomenon is simply reduced to a cell division. Basically it would only be a matter of this: that in the germinal cells of a species which has reached a certain “degree of maturity” a particular “differential division” takes place at some early stage, so that the species-stipes of the sons, instead of succeeding as usual between them (A = A) and equal to those (A) of the mother, they succeed instead [sic] different between them (B, Q and different from those (A) of the mother. (Rosa, 2001, p. 414)

Hence, Rosa considered the environment to be a filter through which suitable forms were preserved and unsuitable forms prevented. Further, he grounded his theory on organism internal features and brought this notion to its extremes. He borrowed the notion of ideoplasm from the Swiss botanist Carl Wilhelm von Nägeli (1817–1891) and used it as the material basis for his concept of morphogenesis and dichotomic branching speciation.^11^ Last, and most importantly, from the strong parallelism between the life of species, the life of individuals, and the combination of characters responsible for form development, Rose deduced that there was a sort of intrinsic limit which set and constrained organisms’ further development. Once organisms reach this limit, the species is bound to become extinct since no further combinations of characteristics are possible.

This idea suggested that evolution was a directed and irreversible process. Rosa supported, in fact, an orthogenetic view of evolution: “if over time a species evolves, the direction of this phylogenetic evolution is determined by the nature of that species (by the nature of its mother cell), and if that species splits into new species, the constitution of these species is determined by the constitution of the mother species” (Rosa, 2001 / [1918], p. 414).

As mentioned, Colosi was part of a broader network of biologists interested in bringing out the architectonical proprieties of form. For instance, in several of his works, Colosi discussed Berg’s theories at length and defined them as essential to his understanding of morphological parallelism.^12^ He wrote, for instance, that “many of those interesting facts, highlighted by BERG and incorporated by him under the name of phylogenetic precessions, simultaneously demonstrate the law of the dissymmetry of the twin groups and the law of the morphological parallelisms. In groups that have established themselves in an earlier period, some characters appear which anticipate similar characters that would appear later in newer groups” (Colosi, 1945, p. 105).

In his 1922 Russian book *Nomogenesis, or Evolution Determined by Law*, which was translated into English in 1926, Berg vehemently attacks the Darwinian model of evolution. In particular, he refused to assign a key power to natural selection and chance in grounding morphogenetic processes and speciation. “The object of the following pages,” Berg wrote in one section of his book, “is to show that the evolution of organisms is the result of certain processes inherent in them which are based upon law. Evolution is Nomo-genesis, development in accordance with definite laws, and not as was believed by Darwin, development due to chance” (Berg, 1926). Berg was interested in the modus operandi of evolution. This proceeded not due to the “vagaries of chance, but the whole process is an orderly one, defined by strict natural law” (ibid., p. ix). Adopting an empiricist approach to evolution, which was shared by all of the practitioners of the bottom-up approach to form, Berg also professed that, in his book “no hypothesis is evolved: facts speak for themselves” (ibid., p. xii).

Furthermore, Colosi deeply appreciated Russian zoologist Michael M. Nowikoff’s morphological research. Nowikoff (1876–1965) investigated the material and structural elements responsible for morphogenesis. For instance, *Foraminifera* shells could indeed be explained by purely mechanical factors and constructional principles such as form generating from the smallest surface area occupiable of a particular material or the search for maximum strength within the material available. However, Nowikoff noticed that these factors were:

nothing more than the expression of the relationship of the inner structure of the clove, i.e. the inherent morphogenetic tendencies with the outside world. These interactions can be very simple: When a drop of the viscous protoplasm takes on a rounded shape, contact with the outside world is minimized. In other cases, this reaction is more complicated, with either internal forces, such as the growth of the cell body and thus of the shell, or external influences, the result of which manifests itself in the solidification of the shell. (Nowikoff, 1953, p. 60)

Form depended on “the internal properties of the organisms, i.e. the highly complex reactions that take place between the components of the organism on the one hand and between the organism and its environment on the other” (ibid.). Besides Colosi, Berg, and Nowikoff, an additional key Italian biologist was keen on tackling the morphological problem: Giuseppe Levi.

## Form, growth, and ageing

Giuseppe Levi was born in Trieste in 1872. After his studies and a year in Berlin at the Institute of Anatomy and Biology directed by German embryologist and zoologist Oscar Hertwig (1849–1922), he became the assistant to the anatomist Giulio Chiarurgi (1859–1944) in Florence in 1899. There, he participated in the first editions of Chiarurgi’s *Treatise on Human Anatomy*. From 1916 onward, he served as a professor of human anatomy at the universities of Sassari, Palermo, and, eventually, Turin. He taught in Turin until 1938 when he was dismissed because of his Jewish origin. After emigrating to Belgium and several vicissitudes because of his participation in anti-fascist resistance, he returned to Turin in 1941 where he worked in a clandestine laboratory with his assistant Rita Levi Montalcini (1909–2012) for about a year. At the end of the war, he was reintegrated in Turin where he died in 1965. Among his students in Turin were the Nobel Prize winners Salvador Luria (1912–1991), Renato Dulbecco (1914–2012), and Rita Levi-Montalcini. Hans Elias (1907–1965) was also his student in 1934; Elias went on to co-found stereology.^13^

The research agendas of Colosi and Giuseppe Levi intertwined several times over the years. One of these was particularly significant. For a book series he was setting up, Colosi asked Levi to write a monograph that dealt with the morphological problem as expressed by Pensa. This monograph was meant to be “a bridge between the cytology of microscopic preparations and the physics of colloids.”^14^ Levi, however, did not accept this invitation because of his “limited knowledge of physics-chemistry”, as he wrote to Colosi. However, he countered Colosi’s offer by proposing a volume of about 200 pages about growth and senescence. This topic, Levi added, “is a subject to which I and my collaborators have made some modest personal contributions and which I could carry out, precisely because it is very personal, without having a library at my disposal.”^15^ Colosi accepted Levi’s proposal with great enthusiasm. Levi began to work on the subject in the complete isolation imposed by World War II.

However, his most important publication was the *Trattato di istologia* [Tratise of Histology], since published in many editions, revisions, and translations. This textbook, as well as his other studies on aging, conferred some different lines and approaches proper to morphological research of the early twentieth century: organicism, the focus on form structure, D’Arcy Thompson’s analyses of form changes, and the morphological investigations of mechanical morphology supported primarily by the German-speaking biologists. In fact, that Levi’s *Tratise of Histology* has been translated and published in various editions is a testament to the profound change that has taken place within morphological research in the first half of the twentieth century. In the first edition (1927), Levi noted that “the renewal movement in Morphology, initiated by the genius of Roux, has in recent years vivified histology as well” (Levi, 1927, p. iv). In the third edition, published in 1946, this vivification of morphological research was transformed into a full “renewal of morphology” based on the correlation between the experimental study of form structure and its connection to the function of a given form.

Levi recognized the central problem that held these aspects together in the structural issue of form inner organization, i.e., in the morphological problem as formulated by Pensa. It is worth quoting it in full from the *Tratise of Histology*:

The constituent substance of living organisms has particular physical and chemical properties, but its most essentially specific character, and which more than any other is connected with the vital manifestations, consists in the structural properties, which have no comparison in the inanimate world; they are summarized in the word organization; by this word is meant the permanent spatial distribution and the physical state of the essential constituents of the living system. There is no life without organization; it changes rapidly when a living being dies, and after some time, it is erased. (Levi, 1946, p. 1)

Organisms are defined, therefore, as complex structured organizations and, as Levi recognized by quoting Przibram, “the characteristic of the living substance is to unite these properties at the same time in a harmonic whole” (ibid.).

Once the definition of organisms was established, Levi developed the purpose and tasks of histology. This discipline should investigate the form-function relationship. This meant that it sought to analyze the origin of this complex relationship and all its possible modifications through time. As he put it, “the contrast between structure and function that instinctively presents itself to our minds is illusory, since structures are never at rest but in continuous becoming; within very small dimensions, these two concepts actually converge” (ibid., p. 2). This statement indicated a deeper point. Form was meant as a changing entity. *Becoming*, i.e. morphogenesis, was what characterized the living beings.^16^ Therefore, form could not be studied statically; instead, it should be studied dynamically, in all its possible development paths and growth dynamics.

To better study the interdependence of form and structure at the microscopic level, Levi expanded on the method of in vitro cultivation. Developed by American biologist Ross Granville Harrison (1870–1959)^17^, the cultivation in vitro of cellular lines was based on a series of procedures to preserve the cell detached from its original organism. The tissue fragments were placed in a hermetically sealed glass cell after being immersed in a small drop of blood plasma taken from an animal of the same species as the tissue to be grown. The culture was maintained at a temperature between 38° and 40 °C. Under these conditions, the tissue finds an environment that allows it to live in conditions not too dissimilar from its usual conditions. As a result, the culture can develop further. For example, French physiologist Alexis Carrel (1873–1944) was able to keep cells from a fragment of chicken embryo alive for almost 30 years.

Levi was a strong supporter of this method. First, it enabled him to study the form and structure of cells while taking into account their possible growth and changes over time. Furthermore, it provided him with much more than a mere snapshot of a single growth moment. Second, tissue cultivations in vitro also provided important insights into the form problem. The possibility of splitting and abstracting cells from an organism implied that “the individual organs and tissues were equipped with a certain degree of functional autonomy” (Levi, 1919, p. 21). Furthermore, the phenomenon of morphogenesis and the different regularities according to which the form-function complex may adapt to a different environment could be observed and examined in-depth. “In these conditions so different from the usual ones”, noted Levi, cells “profoundly modify their form by adapting themselves to the new environment. If the clot is thick, we see them arranged in several layers and take a tapered shape; or they emit very long extensions directed in various planes. If the clot is thin, the cells expand into very thin plates adhering to the lower face of the slide, and then the structure of the protoplasm can be studied at the highest magnification” (ibid., p. 23).

Hence, as science historian Arianna Dröscher noted, “unlike Carrel, Levi also did not claim that the cell itself, if isolated, was immortal. Rather, he emphasized that in his cultures he never succeeded in establishing a line that lived forever. In his experiments, the cells had a ‘limited viability’” (Dröscher, 2018, p. 39).

Fourth, the technique of in vitro cultivation gave technology an extremely important role in the development of morphology: “this method allows to study even with the highest magnifications the cells under the microscope, and to follow the biological manifestations” (Levi, 1919, p. 21). By using such technology to study form structures, Levi aligned himself with the technologically-driven investigations of form in twentieth-century morphology.^18^

These analyses of form and structure led Levi to focus on the topics of growth, its limits, and its biological significance. Explicitly following the morphological tradition of D’Arcy Thompson, he defined growth as a “limited, permanent, and irreversible increase in volume on living material” (Levi, 1946, p. 3). Given this definition, he analyzed the causes responsible for growth and aging. Levi considered aging and death the two mothers of the development of the organism. Unlike the cell, the organism, in fact, cannot live forever.^19^ Cells make up the architecture of the organism. As a result, Levi recognized the importance of the cell system’s autonomy. It, noted Levi, “is conceived not as an aggregate of autonomous particles, but as a unitary mechanism, in which it cannot be imagined [sic], that there are parts of the protoplasm which survive others; all the elementary constituents of the cell are in restricted relations of interdependence” (Levi, Papere & Viale 1933, p. 291). Growth and aging resulted from a system or diagram of forces (as D’Arcy Thompson noted) acted of the organicism. Or as Levi noted, directly quoting Driesch and echoing Colosi and Rosa, “the limit has been marked by the need not to exceed the predetermined goal indicated by the limit size of the soma” (Levi, 1919, p. 30).

This does not mean though that Levi eventually adhered to vitalism, but on the contrary it was Levi’s call to place the research’s emphasis on the architecture and composition of the organism, in effect showing its systemic character. “It is thus demonstrated that the limitation in the growth of animal organisms does not depend on factors inherent in the cells, but on the same causes that regulate the shape and architecture of the organs, of which at the moment we are not able to define, but only to suspect the nature and mechanism of action” (ibid.). Hence, Levi deeply accepted the broader organicism framework and used it as useful strand to support his empirical investigation.

## Conclusion

Over the past decades, we have broadened our understanding of twentieth-century biology. The view that evolutionary biology was shaped by a hyper-selectionist and gene-oriented approach developed during the so-called Modern Synthesis of Evolution has been considerably expanded upon and, in part, abandoned. Several so-far overlooked research traditions have been investigated in-depth, thus providing new insights into the development of twentieth-century life sciences, like the history of evolutionary developmental biology or epigenetics (see for instance (Huneman & Walsh, 2017; Love, 2015)). Although more writing is being produced on this topic, still more efforts are required before a comprehensive overview of how the various theoretical traditions, which featured the nineteenth and twentieth centuries, is reached. This paper intends to contribute substantially to this trend by focusing on the role of Italian-speaking morphology in early twentieth-century evolutionary biology. Looking at the practices and research agenda developed by Colosi and Levi, this paper has thus provided the first necessary elements for understanding the network of biologists investigating on morphology under the organicism framework – in this case, by adding Italian-speaking biologists to this network. This sets the stage for a more complete analysis of the role of morphology within twentieth-century evolutionary biology in the future.^20^

First and foremost, the issue those biologists raised was *methodological*. They loudly requested that morphologists should find an appropriate method for studying form features and the dynamics of form changes through time. As Levi programmatically put it, “for too many years, morphologists have kept away from the study of vital phenomena, as if these were not of their competence, forgetting that the forms that were minutely described by them were governed by mysterious factors, that the methods then available to science did not allow to trace. Morphology then lacked above all methods of investigation. Now, for the first time, we are allowed to analyze the structure of the living protoplasm of higher organisms, to follow the transformations in the locomotion and reproduction of cells, to determine the reactions of the protoplasm to artificially induced stimuli” (Levi, 1919, p.32). As a result, morphologists were convinced that the possibility of contributing to the examination and comprehension of vital phenomena was now possible through an exhaustive re-grounding and reformulation of their methods of investigation.^21^ “The two methods, the descriptive and experimental ones”, Levi added, “must converge to a single end: the investigation of the factors [that governs] forms’ [structures] and their functional meaning, summarized in the denomination of Causal Morphology” (Levi, 1920, p. 66).

Second, through a clear assessment of morphology’s new method, morphologists aspired to be treated as *genuine biological and evolutionary scientists*. In fact, the study of form was seen by all the practitioners as the necessary and most fruitful way to study evolution and discover new evolutionary patterns and mechanisms. What morphology could offer to evolutionary theory was indeed a systemic view. Organisms and organic forms should be studied systemically. This systemic point of view could, in turn, broaden the evolutionist’s analysis, in effect avoiding possible reductionisms. In this regard, Levi admitted that the notion of “structure alone could not suffice to explain the dynamic manifestations of life, just as neither the chemical nor the colloidal properties of living matter can suffice. Life is not bound to a special substance, and not a special reaction, but is a great system of processes and substances which cannot be dissociated; all these properties of organized substance are with perfect harmony adapted to function, from the most elementary to the most complex” (ibid., p. 44). Colosi was also very clear about this point. Only through an in-depth study of the morphology of organisms and their inner architectures were new evolutionary mechanisms, such as hologenesis, detectable.

Third, within this disciplinary and methodological attitude, histology was seen as a central discipline that could help unearth the way in which organisms’ building parts were put together. Levi shared this opinion with other biologists. Among them, he deeply respected and shared Petersen’s form analyses. In his writings, German biologist Hans Petersen (1885–1946) defended an all-encompassing concept of animal form or, as he called it, *Tiergestaltung*^22^. Form was meant as a ”ready-to-use solution to a constructional task” (Petersen, 1922, p. 343). As a result, Levi, and Colosi and Pensa before him, focused on the laws that rule the architecture of organisms. They were keen to show which architectonical composition, i.e., organism, was possible and which rule of composition shaped morphogenetic processes. Levi’s in vitro cultivation was meant to contribute to this process since it provided essential elements to understand how building blocks, which constitute organic forms, can be combined. Furthermore, it provided valuable knowledge to understand the phenomenon of form growth and particularly the differences between organic and technology-driven generated forms. In organisms, cells and organs obtain a specific structure and architecture that cannot be formed outside the organism. As Levi admitted, “these pieces of research show, therefore, that cells isolated from the organism maintain all their original activities; they breathe, feed themselves, eliminate waste products […] sometimes they maintain the specific morphological characters they possessed in the tissue” (Levi, 1919, pp. 27-28). He later added: “In the present state of our knowledge, I believe that masses of elements grown outside the organism, while sometimes maintaining their specific morphological and functional characters, never repeat either the form or the architecture of the organ from which they come” (ibid., p. 29). The study of form opened up the examination of the architectural composition of form and organisms.

This research topic was fully integrated within the organicism movement, despite taking up the language of engineering (biologists spoke of architecture and structure of form). As mentioned, these biologists also aimed at treating the organism as a whole without accepting neither vital forces nor a reductionistic vocabulary. Hence, by calling attention to this research agenda as practiced in Italian-speaking biology, this paper paved the way for a nuanced and more varied picture of the organicism movement in the first half of the twentieth century. In fact, besides dialectical materialism and holistic biology, two of the main strands within organicism, the architectural approach to evolution also had a profound impact on twentieth- and twenty-first-century organicism and, more broadly, on evolutionary biology.^23^ Unlike dialectical materialism, holistic biology (Baedke, 2019), and philosophical anthropological approaches to biology (Jaroš & Brentari, 2022; Gruevska, 2022), the architectural approach to evolution as defined by Italian-speaking biologists did not emerge from broader philosophical frameworks. Rather, it grew out of a meticulous study of the internal organization of form and a strong disappointment with the established methods from morphology. Colosi developed it from his analyses of the parallelisms; Levi based it on the data amassed using in vitro cultivation.

Last, Levi, Colosi, and other biologists used morphology and in vitro cultivation as pieces of evidence against vitalism. As Levi wrote, “the cells of that same organ transported out of the organism and cultivated did not lose anything of the original growth activity; on the contrary, this was reawakened at each transplantation and tended to grow to a relevant extent, as if the inhibiting forces which in the embryo limited, and later paralyzed its activity, had been abruptly eliminated” (Levi, 1919, p. 30). It is thus shown that the limitation in the growth of animal organisms does not depend on factors inherent in the cells, but on the very causes which regulate the form and architecture of the organs, of which we are at present unable to define, but only to suspect the nature and mechanism of action” (ibid.).

Hence, and to conclude, by calling attention to the morphological problem as posed by Italian biologists, this paper has uncovered a network of scientists who worked to uncover the architecture of form. This paves the way for a more nuanced history of early twentieth-century evolutionary biology and morphology.

## Footnotes

1 Although at the turn of the twentieth-century in Germany and other countries terms such as “biology” or “biologist” signaled a particular interest in topics associated with a strong notion of life and/or with what we now understand as ecology, I use these and other terms as the actors analyzed in this paper called themselves. I thank one referee for this.

2 Among others, they were influenced by Ludwig von Bertalanffy and Joseph Woodger. On this context see (Esposito, 2016).

3 On this debate see for instance (Nicholson & Gawne, 2015; Baedke, 2019).

4 On the institutional landscape of Italian biology and morphology see (Tamborini, 2022b; Capocci, 2006; Cassata & Pogliano, 2011; Cassata, 2007; Pogliano, 1999). On the broader institutional landscape of twentieth-century morphology see (Tamborini, 2022b; Rieppel, 2016; Esposito, 2016; Tamborini, 2022a ; Baedke 2019; Bowler, 1996; Nyhart, 1995).

5 See (Mayr, 1980; Tamborini, 2022b; Nyhart,^1995^).

6 See (Driesch,^1919^, ^1936^, ^1898^).

7 Among Italian biologists for instance, biochemistry Filippo Bottazzi (1867–1941) supported a more methodological mechanism. See (Bizzarri, 1936).

8 This was one of the hot topics in morphology during the first decades of the twentieth century. See (Tamborini, 2022b).

9 The call for collaboration between morphological disciplines is quite a classical issue within the history of biology. See, for instance (Tamborini, 2020a; 2022b; Cateforis, Steven, & Steiner, 2019; Cain, 1993; Nickelsen & Krämer, 2016; Nickelsen, 2021; Olechnicka et al., 2018).

10 All translations are mine unless otherwise noted – the debate between Colosi and Levi was mostly in Italian.

11 See also La Vergata, *Introduzione*.

12 D’Arcy Thompson was also a part of this network and supporter of Berg’s ideas. The English translation of Berg’s *Nomogenesis*^1926^featured a forward written by D’Arcy Thompson. In this text, the Scottish polymath indicated that he used the “phrase ‘laws of growth’ in very much the same sense (though with a stronger leaning to the physical side) as Dr. Berg used “Nomogenesis”.

13 See (Grignolio & De Sio, 2009; Piazza, 2018; Bentivoglio, Vercelli & Filogamo, 2006; Dröscher, 2018).

14 Giuseppe Colosi Papers, Università degli studi di Firenze, Biblioteca di Scienze - Polo Sesto Fiorentino, folder Colosi-Levi.

15 Ibid.

16 For more on the notion of morphogenesis in twentieth- and twenty-first-century science, see (Tamborini, 2021).

17 For the history of cultivation in vitro in Italy see (Dröscher, 2018; Grignolio & De Sio, 2009).

18 See (Tamborini, 2022c, 2022b; Liggieri & Tamborini, 2021; Rieppel, 2016).

19 As he put it, “in general, in protozoa and even in bacteria there is no natural death. After a certain period of life, each individual splits into two new individuals of almost equal size, which together contain all the substance of the organism from which they are derived, so nothing is lost and the process continues indefinitely” (Levi, 1919, p. 26).

20 Indeed, Colosi and Levi were part of a larger international biological/morphological community at that time. For this part of the story see (Tamborini, 2022b).

21 This conviction was quite widespread. For a critical account of morphologists’ hope and efforts of grounding their discipline see (Tamborini, 2022b; Dresow, 2020).

22 See also (Tamborini, 2020b). For the broader dynamics of form circulation see (Tamborini, 2022c ).

23 For instance, biologist Stephen Jay Gould was deeply influenced by this approach to evolution (Tamborini, 2020a).
